# Omnivores, Flexitarians, Vegetarians, and Vegans Attach Different Importance to Eleven Motives for Daily Food Choice Decisions: Findings from 5111 UK Adults

**DOI:** 10.3390/foods15040617

**Published:** 2026-02-09

**Authors:** Sara R. Jaeger, Glenn B. H. Andersen, John Prescott

**Affiliations:** 1 Department of Food Science, Aarhus University, 8200 Aarhus N, Denmark; gha@food.au.dk; 2 TasteMatters, Sydney, NSW 1230, Australia; prescott18@gmail.com; 3 Department of Agriculture, Food, Environment and Forestry, University of Florence, 50144 Florence, Italy

**Keywords:** food choice motives, dietary preferences, consumer research, online survey, best-worst scaling

## Abstract

Many initiatives aimed at improving population-wide health or providing food sources that are sustainable and environmentally friendly are focused on a switch from primarily meat-based diets to diets that are more vegetable-based. Building rational approaches to promoting such changes requires an understanding of consumers’ motives for their dietary choices. Aiming to extend prior research, the present study examines eleven food choice motives across nine dietary groups varying in their adoption of diets that are plant-based, from omnivores through meat-reducing flexitarian groups to vegetarian sub-groups and vegans. Using a large population sample and Best–Worst scaling, a novel approach to assessing the relative importance of these motives, we show that the dietary groups are distinguished from one another by a relatively small number of food choice motives. The most substantive of these are Sensory Appeal and Animal Welfare concerns, the former being most characteristic of those consuming meat as part of their diet, and the latter being rated more important by the different vegetarian and vegan groups. Various forms of flexitarian diets are driven by differences in the relative importance of several food choice motives. Generally notable is the finding that, in contrast to previous studies, the importance attached to Health, Weight Control, and Natural Content is not particularly characteristic of any specific dietary approach. The research contributes new fine-grained knowledge about motives for different dietary choices, which can be harnessed for intervention and policy actions.

## 1. Introduction

Understanding dietary choices is increasingly viewed as crucial to addressing both individual and population health issues such as obesity, as well as providing a means to address a range of sustainability concerns, especially those related to high levels of consumption of animal products. For both issues, a central question is the extent to which omnivorous diets high in animal products can be modified by increasing the proportion of vegetables or other substitutes for those animal products [1,2,3,4,5].

Clearly, the foods we consume are, to varying degrees, based on our preferences for their sensory qualities, and a wide variety of factors—genetic, cultural, personality, experiential—are important, individually and in concert with one another, in determining choices for individual food types [6]. However, there is also a broader shift, particularly in developed countries, towards diets increasingly characterized by diversity in the degree of inclusion of animal products. The fact that such shifts have been shown to increase over recent decades [7] suggests that they are largely determined by changes in attitudes, values, or motives. Hence, it is generally assumed that the adoption of a particular attitude towards meat consumption will cause a change in food choice behavior that becomes characteristic of an individual’s overall diet.

One approach to characterizing and differentiating such dietary diversity has been to measure motives for food choices, often using standard questionnaires such as the Food Choice Questionnaire (FCQ) [8,9]. These can provide a snapshot of which motives among many are associated with dietary variations. In turn, understanding motives for consuming a particular diet, or switching to a diet that is wholly or partly vegetarian, can underpin efforts to encourage the adoption of diets that are consistent with health or sustainability initiatives.

### 1.1. Meat Reduction

Since being an omnivore is, in most societies, the dominant dietary type, a crucial distinction is between reasons for meat reduction as opposed to reasons for actively adopting a partial or complete vegetarian/vegan diet, since these may be motivated by entirely different factors. Omnivores have been shown to view meat eating as a natural, or even entitled, behavior that is necessary for health, as well as being pleasurable [10,11,12]. In particular, omnivores may view a switch to a more vegetable-based diet as a loss in food pleasure [13]. Omnivores may also experience inertia in reducing meat intake due to their adherence to a more traditional diet that aligns with accepted social norms [14].

Given these incentives for maintaining a meat-centric diet, the adoption of a more plant-based diet may require competing motivations. Studies to date have suggested that recognition of the health benefits of meat reduction may be important [15,16], although it is acknowledged that the nature of such benefits is still being debated and may not be judged as important by all meat consumers [17]. Nevertheless, higher vegetable consumption appears to be strongly linked to concerns about health [18,19].

Other factors associated with meat reduction have included the high cost of meat, taste preferences, animal welfare concerns, weight control, and a desire to be more environmentally friendly [16]. A recognition of animal welfare issues, in particular, may be linked to dietary change, given the strong possibility that such concerns induce cognitive dissonance [20] regarding meat consumption.

### 1.2. Flexitarian Diet

A reduction in meat in the diet, and associated increased vegetable intake, is generally associated with a flexitarian approach, also referred to as ‘semi-vegetarianism’ [21]. This can refer to a wide range of dietary approaches, all of which involve, as a minimum, a reduction in the consumption of meat—in particular, red meat—to varying degrees [22]. Other flexitarians subscribe to an almost completely vegetarian diet, with exceptions for particular groups of foods, such as fish or shellfish [22,23], an approach that implies the adoption of a dietary strategy over and above a simple reduction in meat intake.

As might be expected given the wide range of dietary content, flexitarians cite a broad range of motivations for their dietary choice, many of which overlap with those cited by meat reducers, vegetarians, and vegans [23]. In fact, it has been noted that treating flexitarians as a unitary group risks ignoring the entirely different motivations behind their dietary choices [23]. Like meat reducers, those who identify as flexitarians cite health concerns as a major motivation for their diet [9,15,22,23]. In addition, like vegetarians, flexitarians also tend to show greater interest in natural products [9,24,25] and are more likely than omnivores to cite ethical motivations for their dietary choices, e.g., agreeing that animals and humans have similarities in emotional and mental capacities [9,24]. General animal welfare concerns are particularly evident in those who have shown the greatest reduction in meat consumption [22].

Different types of flexitarians have been described, based on their primary motivations to adopt that diet. Thus, Sheen et al. [26] identified three distinct flexitarian clusters defined in terms of reasons for meat reduction—health-driven, being cautious about novel food technologies, and being adventurous with respect to animal alternatives—but that were independent of *degree* of meat consumption. Further differentiation of flexitarian consumers has been made in terms of the relative importance of personal (‘egoistic’) motivations such as affordability, sensory appeal, and food safety, as contrasted with ‘prosocial’ motivations related to animal and environmental welfare, the latter being characteristics of those who were more engaged with flexitarianism [7].

### 1.3. Vegetarian and Vegan Diets

Those with a fully vegetarian diet differ from omnivores and meat-reducers/flexitarians on a number of different motives, including an increased emphasis on the natural content of foods and on environmental concerns [14,25,27,28,29]. However, there is strong evidence that the key differentiator is the degree of animal welfare concerns [10,14,15,23,25,29,30].

However, as with flexitarians, there also appear to be different individual motivations for adopting vegetarianism, or at least different emphases on important motives such as health and ethical (animal welfare) concerns [31]. The degree of emphasis on animal ethics issues is also apparent in distinguishing vegetarians from vegans, the latter having stronger motives based on animal rights, which are then applied to all animal products, including eggs and dairy products [30].

In fact, it is not clear whether distinctions between meat reducers, flexitarians, vegetarians, and vegans represent actual motivational subgroups or the increasing importance of a small number of individual motives, in particular animal welfare. Based on responses to an Animal Attitude Scale, De Backer and Hudders [32] were able to differentiate omnivores from flexitarians, and flexitarians from vegetarians, in terms of animal-related concerns. They concluded not only that the degree of concern about animal welfare was directly related to the degree of meat elimination from the diet, but also that increasing ethical concerns might be the motivator for flexitarians to transition to vegetarianism. Similarly, it has been suggested that those flexitarians showing the largest meat reduction, itself associated with animal welfare concerns, are the most likely to later adopt vegetarian or vegan diets [22].

## 2. Research Aim and Empirical Strategy

The aim of the present research is to provide a fine-grained examination of the relative importance of a standard set of food choice motives that encompasses a wide range of reasons for maintaining or adopting various diets. Specifically, the present study extends previous research by examining eleven food choice motives in nine dietary groups varying in their adoption of diets that are plant-based, from omnivores through meat-reducing flexitarian groups to vegetarian sub-groups and vegans.

Access to a large consumer sample (*n* = 5111 UK adults) enabled us to differentiate multiple diet sub-groups, especially within the broad flexitarian category, but also within different vegetarian sub-groups, and thus compare motives for quite specific dietary types. This extended previous research where many studies were based on relatively small sample sizes or had compared dietary groups in very ‘broad-brush’ terms, e.g., omnivores v. flexitarians v. vegetarians.

Consistent with the overall research aim, we sought to determine the extent to which sub-groups were motivationally distinct from one another or, as some research has suggested, whether they are linked by, for example, differing degrees of emphasis on a motive such as animal welfare. In turn, such data may provide the basis of more targeted approaches to encouraging the take-up of health and/or sustainability goals.

In the present study, motives for dietary choices were measured using an FCQ. The original FCQ by Steptoe, Pollard and Wardle [8] covered nine motives across 36 questions. Later variations have expanded the original list of motives by including ethical, religious, and sustainable motives in greater detail [33,34]. Here, a variant of the 11-factor single-item FCQ from Onwezen et al. [35] was used because it represented a compromise that included eight of the nine motives from Steptoe, Pollard and Wardle [8] (excluding mood, since this relates very much to short-term impacts on food choices) and expanded on these for ethical and sustainable motives, clearly more relevant for long-term dietary choices.

A novel methodological aspect to this study was the use of Best–Worst scaling (BWS; specifically Case-1 BWS) rather than the rating scales that are typically used to measure the relative importance of motives [8,33,34,36]. As a choice-based task, Case-1 BWS helps reduce respondent monotony in longer surveys where rating scales are common. In addition, BWS provides data that has proven measurement properties, which, depending on data analysis, can be either interval or ratio [37,38]. In contrast, data from categorical rating scales are usually considered an ordinal-interval hybrid [39] and, more importantly, they offer less discrimination between the BWS objects of interest than Case-1 BWS scaling [37]. This is significant because earlier FCQ studies have shown modest differences in mean ratings across food choice factors (e.g., [36]), suggesting that social desirability bias may affect ratings of some motives. The trade-off approach enforced by BWS prevents this. Finally, it is possible to assess the consistency of each participant’s BWS responses [40] and exclude participants with poor FCQ data quality prior to data analysis. A focus on data quality in online surveys has become paramount due to the risk of inattentive respondents and fraudulent survey bots [41,42].

## 3. Materials and Methods

### 3.1. Data, Dietary Groups, and Participants

To facilitate, per the research aim, a comparison of nine dietary groups, access to a large consumer sample was required, and this was achieved by pooling data from four previously conducted online surveys (Table 1), which were all on the general topic of food-related consumer attitudes and behavior. Beyond methodological efficiency, this approach reflects sustainability awareness by maximizing the scientific value of existing data and reducing the need for additional data collection.

These various surveys had different aims and objectives, and, in turn, different criteria for participant inclusion and exclusion. Of particular relevance for this research was the self-declared dietary group, which was measured using a question from De Backer and Hudders [32] with nine predefined options covering omnivores, several flexitarian and vegetarian subtypes, and vegans (Table 2). Omnivores and flexitarians were always eligible, but vegetarians and vegans were excluded from two of the studies (Table 1). This led to a further skewing of these less frequent diet groups in the total sample. Collectively, the three vegetarian sub-groups and the vegan group accounted for 11.8% of the total sample. The omnivore group accounted for 64.8%. Thus, the sample size in the different diet groups varied considerably (Table 2).

The participants in all four online surveys were adults (18–69 years old) residing in the United Kingdom (UK) who had self-registered with ISO 20252-certified [43] commercial market research providers, as they were interested in participating in survey research. Each of the participant samples in the four studies was diverse across various characteristics, such as education, household income, and household composition.

Table 3 presents a summary of participant characteristics for all participants and by self-declared diet group (*n* = 9). Table S1 of the Supplementary Materials contains participant summaries by study.

### 3.2. Measurement of Food Choice Motives

#### 3.2.1. Food Choice Motives

Based on the 11-factor single-item FCQ from Onwezen et al. [35], the 11 food choice motives and wordings were as follows: Health (‘is healthy’), Freshness (‘is fresh’), Convenience (‘is convenient (in buying and preparing)’), Sensory Appeal (‘is tasty’), Natural Content (‘is natural’), Price (‘is affordable’), Weight Control (‘helps me control my weight’), Familiarity (‘is familiar’), Environmental Friendliness (‘is environmentally friendly’), Social Justice (‘is socially sustainable (e.g., fair-trade)’), and Animal Welfare (‘is animal friendly’). The only slight wording difference between the studies occurred for Study 4, where the Social Justice motive (‘is socially sustainable, e.g., fair-trade’) was replaced with ‘is fair-trade’.

#### 3.2.2. Case-1 Best–Worst Scaling

**Figure 1:**
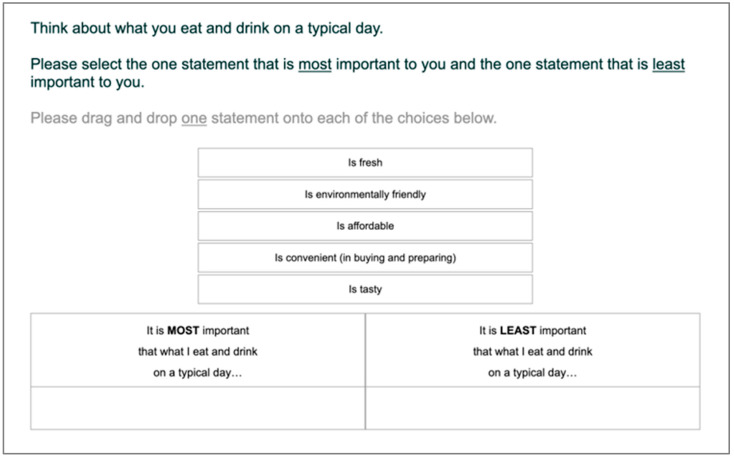
Case-1 Best–Worst scaling (BWS) task used to measure the importance of food choice motives. The instructions to participants were as follows: “Think about what you eat and drink on a typical day. Please select the one statement that is most important to you and the one statement that is least important to you. Please drag and drop one statement onto each of the choices below”. One of the eleven choice sets is shown above.

Case-1 Best–Worst scaling (BWS; [37,47]) was used in this research (Figure 1) to determine the relative importance of the food-choice motives. Also known as MaxDiff (or object case BWS), Case-1 BWS extends the method of paired comparisons [48] to multiple choices. Because respondents are repeatedly required to select the most and least important factors within choice sets comprising subsets of factors, the resulting importance scores express how strongly a factor is preferred relative to the others included in the design, but not whether it is important in absolute terms.

BWS implementation was according to a balanced incomplete block design [49], which defined 11 choice sets, each containing 5 choice options (i.e., food choice motives) (refer to Table S2 of the Supplementary Materials for the design). Participants were instructed to think about what they eat and drink on a typical day and select the most (best) and the least (worst) important factor. The food choice motives were randomized within choice sets, and the presentation order of sets was randomized across participants. This helped to reduce presentation order bias.

### 3.3. Participant Profiling and Data Collection

Standard demographic and socioeconomic questions were used for participant profiling (Table 3). In addition, three other measures were collected to extend the characterization of participants. Self-rated life satisfaction [46] was collected on a 10-point category scale (1 = ‘Not satisfied at all’ to 10 = ‘Extremely satisfied’). Overall general health was assessed with the first item of the short form Health Survey [45], in which participants rated on a five-point category scale (1 = ‘Excellent’ to 5 = ‘Poor’). The 10-item Food Neophobia Scale (FNS) was administered with fully labeled 7-point Likert scales (1 = ‘disagree strongly’ to 7 = ‘agree strongly’) [44]. Food neophobia is a personality trait scale characterized by the avoidance of novel or otherwise arousing foods and beverages.

The surveys were conducted in English, using British spelling. The participants completed the survey online using a mobile phone, desktop, or laptop computer. The four studies were conducted between the end of 2023 and the end of 2025, each lasting a few months. In each study, the main data collection was initiated by a revision of test links and the evaluation of responses from ~10% of the total sample in each study, to ensure that the survey performed as expected.

### 3.4. Data Cleaning and Analysis

All analyses were performed using R programming language version 4.5.1 (R Core Team, Vienna, Austria, 2025). The significance level for inference testing was 5%.

#### 3.4.1. Data Cleaning

Following the recommendations of Jaeger and Cardello [50], data integrity and quality checks were conducted before data analysis. Examples of exclusions were multiple survey completions from the same IP address, fast survey completions (less than one-third of the median time), and flatlining on a 10-item attitude scale battery (i.e., providing the same answer to all ten items). Because the data for Studies 1–4 came from different studies, other aspects of the preliminary data cleaning protocols were study-specific (refer to Parts Text S1–Text S4 of the Supplementary Materials for full details).

In all studies, the Error Variance Normalized (ErrVarNorm, EVN) index [40] was also used for data cleaning purposes. EVN is a normalized extension of the ErrVar index [37] and measures the quality of individual participant’s Case-1 BWS data, while also serving as a proxy for overall survey data quality [40,51]. The ErrVar index, shown in the numerator in Equation (1), is calculated by squaring the B–W score for each object and then summing the squares (with *p* being the number of objects seen and x_i_ being the B–W score for object *i*). B-W scores are the ‘best’ frequency counts (i.e., the number of times the object has been selected as ‘best’) minus the ‘worst’ frequency counts (i.e., the number of times the object has been selected as ‘worst’). Since a consistent respondent will tend to select the same ‘best’ and ‘worst’ options every time they are available, higher ErrVar values signify greater response consistency. Lower ErrVar values signify less consistency [37]. EVN is obtained by dividing ErrVar by its observed maximum value among the respondents per Equation 1. Using the more conservative of the two exclusion thresholds proposed by Llobell, Choisy, Chheang and Jaeger [40], all participants with EVN < 0.5 were excluded before data analysis.

(1) $$ErrVarNorm=\frac{\sum_{i=1}^{p}x_{i}^{2}}{Max_{r=1,\ldots ,m}\{\sum_{i=1}^{p}x_{i}^{2}\}},$$

#### 3.4.2. Data Analysis

The data were first analyzed at the aggregate level using the responses from all participants, and then analyzed by diet group. The same set of procedures was used for the two sets of analyses.

The FCQ data from Case-1 BWS were analyzed by counting the number of times each food choice motive was chosen as most important (best, B) and the number of times it was chosen as least important (worst, W) [37,52]. These were used to calculate individual Best–Worst (B–W) scores. The balanced incomplete block design employed in this study produced individual B–W scores ranging from −5 to +5. A score of +5 means that, across its appearance in 5 of the 11 choice tasks, the participant always chose that food choice factor as best and never as worst. A score of −5 means that the participant always chose the focal food choice motive as worst and never as best. A positive B–W score indicates that a food choice motive was selected as best more often than as worst, while negative values indicate that it was selected as worst more often than as best. Therefore, B–W scores close to zero indicate that the statement was rarely selected at all (or equally frequently selected as best and worst), reflecting a neutral importance relative to the other food choice motives.

In addition to B–W scores, best and worst sums were used to calculate object importance scores (Equation (2)), which, according to Marley and Louviere [38] has ratio-level scaling properties. For example, if Motive 1 has the importance value N1 and Motive 2 has the importance value N2, then N1/N2 expresses the number of times that Motive 1 is more or less important than Motive 2. To improve interpretability, the relative importance of different food choice motives was expressed as relative importance scores (Equation (3)) or proportional importance scores (Equation (4)). In these equations, $B_{i}$ and $W_{i}$ are the numbers of “best” and “worst” selections for individual I among N individuals; importance is the importance score for motive I; and where $\max_{j}\left(Importance\;{score}_{j}\right)$ and $\sum_{j=1}^{K}Importance\;{score}_{j}$ denote, respectively, the maximum and the sum of the importance scores across all motives j.

Inferential statistics were obtained from linear mixed effects models [53] using the B–W scores as the dependent variable. Two models were fitted. An overall model with motive as the only fixed factor (and a random intercept for participants) was used to assess overall differences. Pairwise comparisons of estimated marginal means were evaluated with Tukey’s HSD to provide the overall groupings [54]. An interaction model with diet group, motive, and their interaction as fixed factors and a random participant intercept was used to assess diet-specific patterns. Differences among motives within diet groups and differences among diet groups within each motive were examined using Tukey’s HSD, with lower- and upper-case letters, respectively, indicating significant contrasts.

Principal Components Analysis (PCA) was performed on the matrix of B–W scores defined by the 11 food choice motives and nine diet groups. The covariance matrix was calculated on centered data (by motive). Loadings representing motive variables were scaled according to the default settings of the fviz_pca_biplot() function in the *factoextra* package [55] to match the standard biplot representation used in PCA visualization. The supplementary demographic variables, represented as correlations with the principal components, were normalized to unit length before plotting to ensure a clear and consistent visualization.

For demographic characteristics, categorical and continuous variables were compared using chi-squared tests and one-way ANOVA, respectively. Individual food neophobia scores were calculated by aggregating responses across the ten items, accounting for reverse-coded items. The scale’s internal consistency was high, with Cronbach’s alpha exceeding 0.7 in all studies (0.88 in Study 1; 0.89 in Study 2; 0.89 in Study 3; 0.89 in Study 4).

(2) $$Importance\;score=\sqrt{\frac{\sum_{i}^{N}B_{i}}{\sum_{i}^{N}W_{i}}}$$

(3) $$Relative\;importance\;{score}_{i}=\frac{{Importance\;score}_{i}}{\max_{j}\left(Importance\;{score}_{j}\right)}\times \;100$$

(4) $$Share\;of\;importance\;{score}_{i}=\frac{{Importance\;score}_{i}}{\sum_{j=1}^{K}Importance\;{score}_{j}}\times \;100$$

## 4. Results

### 4.1. Importance Scores of the Aggregate Sample

Table 4 shows the results for all 5111 participants. Sensory Appeal (‘is tasty’) was most important for daily food choice decisions, followed by Health (‘is healthy’), Freshness (‘is fresh’), and Price (‘is affordable’). The relative importance scores revealed that Sensory Appeal was about 1.8 times as important as Health and Freshness (100:57, 100:55), and about 2.3 times as important as Price. The mean B–W scores for Natural Content, Animal Welfare, and Convenience were significantly different, but were all around zero, indicating that these food choice motives were of about average importance in the aggregate consumer sample. The four least important motives were Environmental Friendliness (‘is environmentally friendly’), Weight Control (‘helps me control my weight’), Familiarity (‘is familiar’), and Social Justice (‘is socially sustainable (e.g., fair-trade)’). Sensory Appeal was 11.1 times more important than Social Justice (100/9), the significantly least important motive. The standard deviations for the mean B–W scores ranged between 1.7 and 2.6, revealing heterogeneity in the importance of all 11 food choice factors, but less for Natural Content and more for Weight Control (histograms of overall and by diet group B-W scores are found in Supplementary Materials Figure S1 and Figure S2, respectively).

### 4.2. B–W Scores by Diet Group

**Figure 2:**
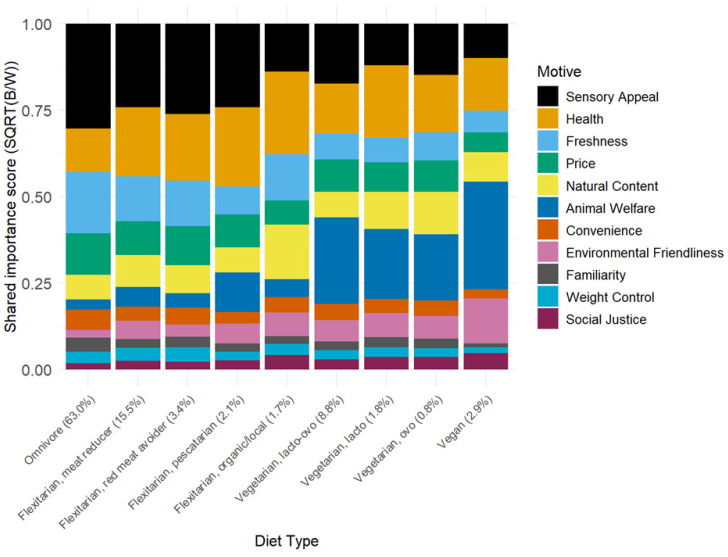
Stacked bar plot of share of importance scores for 11 food choice motives in nine diet groups (UK adults, *n* = 5111). Elicited using Case-1 Best–Worst scaling (BWS) and calculated as √(B/W) following Equation (4).

Table 5 shows the mean B–W scores for the 11 food choice motives in the nine diet groups. Tables of standard deviations and pairwise comparisons of B–W scores within food choice motives across diet groups can be found in the Supplementary Materials (Tables S3 and S4). Figure 2 supplements Table 5 and shows the share of importance as stacked bar plots for each of the nine diet groups (see also Supplementary Material Figure S3 for corresponding funnel plots of relative importance scores across the 9 diet groups).

For Omnivores, the largest dietary group in the study (63.0%), Sensory Appeal (2.6) was significantly most important for daily food choice decisions, followed by Freshness (1.4), Price (1.3), and Health (1.1). Freshness and Price did not differ significantly in mean importance. Natural Content (0.2) also had slightly above average importance. Least important for Omnivores were the three ethically oriented motives—Animal Welfare (−0.9), Environmental Friendliness (−1.5), and Social Justice (−2.1)—together with Weight Control (−1.3). These four motives differed significantly in importance, with Social Justice being significantly least important. Sensory Appeal was significantly more important to Omnivores, and Environmental Friendliness was significantly less important than for any other dietary group.

For the Meat Reducer flexitarian subgroup (15.5%), Sensory Appeal (1.8) and Health (1.8) were the most important food choice motives, with B–W scores that were not significantly different. The third most important in the Meat Reducer group was Freshness (1.0), followed by Price (0.8) and Natural Content (0.6), with no significant difference in importance between Freshness and Price, and Price and Natural Content. Animal Welfare (−0.1) and Environmental Friendliness (−0.3) were similarly important, and significantly more important than the remaining three motives—Weight Control, Familiarity, and Social Justice.

Figure 2 suggests that the patterns of importance scores among flexitarians identifying as Meat Reducer (15.5%) and Red Meat Avoider (3.4%) are quite similar. This held insofar as the rank order of motives by B–W scores with above-average importance, which was similar in the two diet groups. The motives with below-average importance were also the same in the two groups. In the Red Meat Avoider group, the motives Animal Welfare, Environmental Friendliness, and Convenience did not differ significantly, whereas in the Meat Reducer group, Convenience was relatively less important.

Among the flexitarians identifying as Pescatarians (2.1%), Sensory Appeal (1.9) was the most important motive, closely followed by Health (1.7). However, Animal Welfare (1.2) was the third most important motive, being significantly more important than Environmental Friendliness (0.0) but not significantly more important than Price (0.8), Freshness (0.5), and Natural Content (0.3).

In the smallest of the four flexitarian groups (1.7%), where organic and local foods were prioritized, Health (2.1) rather than Sensory Appeal (1.2) was the most important food choice motive. Natural Content (1.3) and Freshness (1.0) had B–W scores that were not significantly different from Sensory Appeal. Environmental Friendliness (0.0) and Animal Welfare (−0.3) had a similar importance as Price (0.1) but were both significantly less important than the motives with above-average scores.

There were three vegetarian diet groups, of which lacto-ovo vegetarian was the largest (8.8%). In this group, in which people included both milk and egg products in their diets, the most significant food choice motive was Animal Welfare (2.3), followed by Sensory Appeal (1.5) and Health (1.2). This revealed a majorly different pattern of motives than observed among Omnivores, where Sensory Appeal was more important than Animal Welfare (mean diff. = 3.5) compared to the lacto-ovo vegetarian group (mean diff. = −0.8). Environmental Friendliness (−0.1) was significantly less important than Animal Welfare (2.3) in this dietary group. The importance placed on Natural Content was the same as observed for the Omnivore group. For the lacto-vegetarian group (1.8%) (i.e., who included milk but avoided eggs), Health (2.2) and Animal Welfare (1.5) were not statistically significantly different in importance. Sensory Appeal (0.9) and Natural Content (0.7), Price (0.4), Freshness (0.1), and Environmental Friendliness (0.1) were the next most important motives with B–W scores above average, which did not differ significantly. In a similar vein to what was observed in the lacto-ovo vegetarian group, Social Justice, Weight Control, Familiarity, and Convenience had below-average importance, and they did not differ significantly. The PCA (Figure 2) suggested that the ovo-vegetarian group (0.8%) was similar to the lacto-vegetarian group.

Vegans (2.9%) were notably different from all the other dietary groups in the importance placed on the 11 food choice motives. While similar to the three vegetarian groups in placing most importance on Animal Welfare (3.1), Environmental Friendliness (1.3) was much more important in this group than observed in all other dietary groups, and of the same importance as that placed on Health (1.3). Moreover, Sensory Appeal (0.5) was less valued than in any of the other dietary groups, but not significantly different from groups lacto-vegetarian, ovo-vegetarian, and flexitarian organic/local. Otherwise, the vegans resembled the lacto-vegetarian and ovo-vegetarian groups.

**Figure 3:**
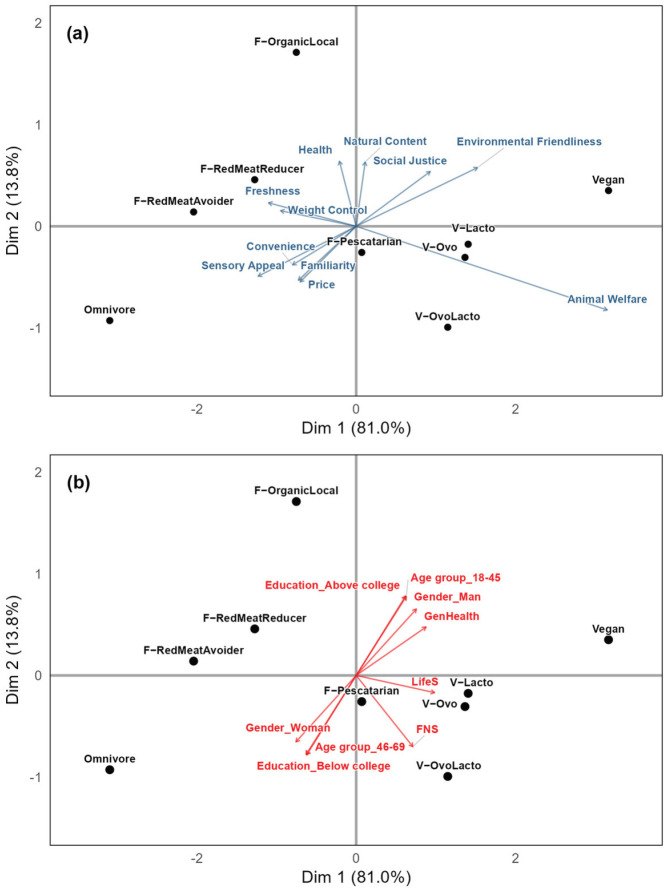
Biplot of the first two dimensions from Principal Components Analysis (PCA) of Best–Worst (B-W) scores of the 11 food choice motives in nine diet groups (black font, circles). The top plot (**a**) displays the loadings of the motives (blue). Bottom plot (**b**) displays the same analysis, but with the gender, age group, education, food neophobia score (FNS), life satisfaction (LifeS), and overall general health (GenHealth) included as supplementary variables represented as vectors (red). Diet groups were “omnivore” (Omnivore); “flexitarian, meat reducer” (F−RedMeatReducer); “flexitarian, red meat avoider” (F−RedMeatAvoider); “flexitarian, pescatarian” (F−Pescatarian); “flexitarian, organic/local” (F−OrganicLocal); “vegetarian, ovo” (V−Ovo); “vegetarian, lacto” (V−Lacto); “vegetarian, lacto-ovo” (V−OvoLacto); and “vegan” (Vegan).

A PCA of the proportional importance values for the 11 food choice factors in the nine diet groups helped to obtain a succinct summary of the similarities and differences between the nine dietary groups (Figure 3a). The first dimension (PC1, 81.0%) separated Omnivores and Vegans and was strongly driven by the difference in importance placed on Animal Welfare by the two groups. The three vegetarian groups, like the Vegans, were located on the positive side of PC1 but in less extreme positions. The flexitarian subgroups Meat Reducer and Red Meat Avoider, which assigned similar importance to the 11 food choice factors (Table 5), were positioned near each other, and on the negative side of PC1, where the Omnivore group was also located. The Organic/Local Flexitarian group was located on the negative pole of the second dimension (PC2, 13.8%) and was more strongly characterized by higher importance being attached to Health and Natural Content.

Figure 3b shows the inclusion of supplementary variables from Table 1 in the PCA. The plot for diet groups and food choice motives is identical to Figure 3a, but the motives are not shown to reduce visual clutter. The supplementary variables (red arrows for gender, age group, level of educational attainment, degree of food neophobia (FNS), overall general health (GenHealth), and life satisfaction (LifeS)) showed a positive correlation between the younger age group of participants (18–45 years old), male gender, and higher educational attainment. The direction of the arrows indicated that these participant traits tended to be more dominant in the Vegan and Organic/Local Flexitarian groups. Self-rated overall general health was also positively correlated with these participant characteristics. The degree of food neophobia and life satisfaction were not strongly correlated with the dietary groups. The Organic/Local Flexitarian group was the least food neophobic and most satisfied with life, on average. There was a tendency for vegetarians and vegans to rate life satisfaction higher than their meat-eating counterparts.

## 5. Discussion

### 5.1. What Does the Relative Importance Assigned to Food Choice Motives Reveal About the Changes Required to Move Towards More Sustainable Food Practices?

#### 5.1.1. Aggregate-Level Results

Before considering the implications of the present findings, we first compare them to past studies to assess their validity. In the aggregate sample of UK adults (*n* = 5111), Sensory Appeal was the most important food choice motive (Figure 2), and almost twice as important as Health and Freshness, the second and third most important motives, respectively. Price was the fourth most important. Compared to the motives capturing Animal Welfare, Convenience, Environmental Friendliness, Familiarity, Weight Control, and Social Justice, Sensory Appeal was 5–10 times more important. Because Case-1 BWS reveals the relative (rather than absolute) importance of the studied food choice factors, comparisons with studies where food choice motive importance was rated directly become qualitative. For example, Jaeger et al. [56] collected data from 1514 UK adults (vegetarians and vegans excluded), using, with two exceptions (Mood (‘is a way of monitoring my mood (e.g., a good feeling or coping with stress’) was included; Freshness was excluded), the same 11 food choice motives as the present study. They reported that the mean importance scores ranged from 3.9 (Mood) to 5.5 (Sensory Appeal) (1 = ‘not at all important’ to 7 = ‘very important’). However, by rank order, the top 4 motives were the same as found here, as were the bottom-5 motives. Markovina, Stewart–Knox, Rankin, Gibney, de Almeida, Fischer, Kuznesof, Poínhos, Panzone, and Frewer [36] also measured ratings of importance and, in a sample of 1061 UK consumers, identified Sensory Appeal (3.59), Price (3.50) and Health (3.33) as the three most important motives (1 = ‘not at all important’ to 5 = ‘extremely important’). This aligns with the current study, where Freshness, which was not included as a food choice motive in Markovina et al., is disregarded. The three least important motives—Weight Control (3.03), Ethical Concern (2.67), and Familiarity (2.60)—also matched the current study. Because Markovina et al. [36] used the 9-factor FCQ by Steptoe, Pollard, and Wardle [8], Ethical Concern was a single motive rather than separate motives for Environmental Friendliness, Animal Welfare, and Social Justice.

#### 5.1.2. Diet Group Comparisons

The characterization by food choice motives revealed clear distinctions between the dietary groups. While there was substantial overlap across groups, especially among those motives that were selected as relatively less important, it is instructive to examine those motives that had noticeably higher B–W scores. Thus, Sensory Appeal, while still somewhat important to most vegetarian groups (the exception being vegans), was given the highest score across all groups that still ate animal proteins, especially omnivores, but also all flexitarian groups. Those still eating meat to varying degrees also tended to select freshness as highly important, perhaps indicative of their awareness of the microbiological issues associated with meat that is not fresh.

As expected, based on previous research, the relative importance of Animal Welfare was clearly evident in vegetarian groups. Previous studies had suggested that increasing Animal Welfare might characterize the various stages of meat reduction from omnivore to flexitarianism to vegetarianism [9,22,24]. In the present data, however, vegetarian groups were distinguished from all others in the strength of this motive, although pescatarians stood out among flexitarian subgroups as also giving a higher score, perhaps indicative of a special status accorded to welfare concerns regarding mammals. Again, as expected, higher importance on Animal Welfare was evident for Vegans. Another standout dietary group was the Organic/Local flexitarians, who alone, and unsurprisingly, gave relatively high importance to Natural Content. Again, this does not support the findings from previous studies suggesting the broad importance of Natural Content [9,24,25]. One reason for this may be the greater discrimination between flexitarian and vegetarian sub-groups in the present study.

In contrast, despite suggestions that the health benefits of meat reduction may be important [15,16,18,19,22], Health was a relatively poor predictor of group differences, being both in the top three motives, as well as receiving a high share of importance scores, for all dietary groups (Table 5, Figure 2). This perhaps lends credence to the idea that there are conflicting views among different dietary groups about the benefits of meat reduction [17]. Several other food choice motives were evaluated as relatively unimportant irrespective of diet group: Convenience, Familiarity, Social Justice, and Weight Control. In the case of Weight Control, this is surprising, since it has previously been linked to meat reduction in the diet [16].

Overall, these data extend previous research, showing that a relatively small number of food choice motives—Animal Welfare, chief among them—distinguish dietary groups from one another. Interestingly, it was not evident that a reduction in meat consumption is consciously associated with increasing Animal Welfare concerns. Rather, this emerged as a characteristic primarily of the various types of vegetarianism. Moreover, although it might be expected that Animal Welfare concerns would be linked to other motives related to social consciousness, such as Environmental Friendliness and Social Justice, this was also not apparent, except among Vegans. Vegans were the only group, even when compared to other vegetarian groups, to give a high importance to Environmental Friendliness. This suggests that Vegans’ motives for their diet are both quantitatively (Animal Welfare) and qualitatively (Environmental Friendliness) distinct from other vegetarian groups.

We also characterize the different dietary groups in terms of three variables that may potentially be influenced by diet. Food neophobia (FN) and measures of overall health [57,58] have both been linked to diet quality, which, in turn, is associated with self-reported health [59]. The possibility, therefore, exists that global dietary choices may be linked to such measures. In fact, there were significant differences in each of these measures across the dietary groups, although there were few indications that these differences were aligned with the specific dietary divisions studied here. Compared to other groups, Organic/Local Flexitarians were noticeably lower in FN and higher in life satisfaction, both of which could conceivably relate to a higher interest in food generally [60] that might partly underlie this type of dietary focus. Vegans scored lowest in overall health, perhaps consistent with reports of some health issues, including vitamin deficiencies, with this type of diet [61], although the small number of participants in this group might mitigate against a definitive conclusion.

Food consumption patterns—omnivore, flexitarian, vegetarian, vegan—may also be linked to more broadly defined categories rooted in personality and personal identity. Meat consumption is negatively related to openness and agreeableness personality characteristics [62], two of the dimensions of the “Big Five” personality theory [63]. In fact, these same dimensions also discriminate between vegetarians and vegans, with the latter being higher along both dimensions [64]. There is evidence too that being an omnivore, a flexitarian, a vegetarian, or a vegan is associated with a specific personal social identity that may direct dietary behaviors over and above more specific motivation factors such as those examined here [65,66]. One clear implication for future research is that characterizing such overarching identities may be important, especially in those cases where individual motives may not distinguish specific dietary patterns.

### 5.2. Limitations and Suggestions for Future Research

#### 5.2.1. Sample, Diet Groups and Food Choice Motives

Despite this study collected data from more than 5000 UK adults, most dietary groups were very infrequently represented in the aggregate sample. Only two groups—Omnivores and Meat Reducer Flexitarians—comprised more than 15% of participants. Four diet groups were each represented by less than 2.5% of participants, and the smallest group comprised only 42 people (0.8%) (ovo-vegetarian). Per Table 1, vegans and/or vegetarians were excluded from three of the four earlier surveys that contributed data to this research. This was a consequence of the four studies having different primary purposes not related to the present research, thereby reducing the number of participants in the infrequent dietary groups. Yet, even if the frequency distribution of the diet groups had been similar to that of the study where all diet groups were eligible for participation, the expected size of the ovo-vegetarian group would have remained below 80 people (Supplementary Material Table S5). Thus, without disproportionate sampling in which the infrequent diet groups were over-recruited, and Omnivores were under-recruited, the very different dietary group sizes would have remained. We decided to stop collecting data when all diet groups had more than 40 participants, since Moskowitz [67] identified the 40–50 range as where stable group estimates tend to emerge in sensory and consumer research. For this reason, we also did not collapse several diet groups into a single group (e.g., merging the three vegetarian groups into one).

Nonetheless, it cannot be ruled out that the results for the infrequent diet groups are less robust (and generalizable) than for the larger groups, and additional data should be collected. Until such a time, it would be prudent to consider the results for the ovo-vegetarian group as preliminary. Additional data are also expected to help correct for the unexpected gender distribution in the vegan group, which comprised 63% men. While the gender gap seems to be reducing, other studies indicate that women are overrepresented among UK vegans [68]. The most likely explanation for the gender discrepancy in the present research is that quota sampling (50% men, 50% women) was imposed in the studies that included vegans and vegetarians. This was performed to satisfy experimental conditions in parts of the surveys that were not connected to the present study’s aims. Because the gender balance in the vegan group was skewed relative to what would be expected if the aggregate sample were nationally representative of the UK, the results for Vegans warrant some caution. A supplementary analysis comparing the 54 women and 92 men in the vegan group revealed several differences in the importance of the 11 food choice factors (Supplementary Material Table S6). Animal Welfare was the most important motive for both genders, but Sensory Appeal and Price were less important to men than women, while Environmental Friendliness and Naturalness were more important. Overall, women integrated their beliefs about Animal Welfare with motives typically given greater importance, whereas men did so to a lesser extent.

The dominance of the Omnivore group, which comprised 63% of all participants, was also a potential cause for concern since, because of its size, it could have been more heterogeneous than smaller diet groups. However, this did not appear to be the case based on the distributions of individual-level B–W scores for the 11 food choice motives (Supplementary Material Figure S2). The visual inspection of the plots did not indicate that the degree of heterogeneity among Omnivores was markedly different from that of any of the other dietary groups, for any motive.

The limitation that the sample was not representative of the UK population, due to the exclusion criteria applied during participant recruitment, is also explicitly acknowledged. If additional data are collected, per the suggestion above, it would be prudent to seek a nationally representative sample. However, as also noted earlier, this risks resulting in low numbers of participants in some diet groups due to their apparent infrequency among UK adults. Obtaining a “perfect” sample is thus not straightforward.

The dietary groups included in this research were based on the classification scheme by De Backer and Hudders [32], which was appropriate given the need for knowledge to help advance the sustainable dietary transition. However, additional insights may have been gained by including a greater range of dietary patterns (e.g., low/no salt, low/no fat, low/no sugar, gluten/wheat free, lactose/milk free, etc.) or others such as ‘snacking’, ‘convenience, red meat and alcohol’, ‘dieting’ and ‘traditional English’ [69,70]. Tentatively, this could have revealed whether participants following diets not directly related to the inclusion/exclusion of animal-based or -derived foods had patterns of food choice motive importance values similar to those studied here. If such matches were found, they could be used to reach consumer groups that do not identify with the labels used by De Backer and Hudders [32]. For example, people who identify as ‘dieters’ may resemble Red Meat Avoider Flexitarians without identifying as such. This is important since public health campaigns that use labels that people relate to are more likely to be effective.

It remains an open question whether the present findings generalize beyond the UK. If diet group shapes people’s daily food choice motives more strongly than national food cultures, then it is probable that the results will be cross-culturally stable. Conversely, if nationality and national food cultures exert greater influence, cross-cultural differences would be expected. National differences in the importance placed on food choice factors have been documented [36,60]. Based on the evidence to date, it seems possible that dietary group/identity and nationality will influence the importance placed on different food choice motives, suggesting that similar data should be collected in other countries and analyzed. If such work were to be conducted, it would be appropriate to consider whether additional food motives should be included. Religion has the potential to influence food choices [34], as do mood [8] and many other factors [33,71]. For the empirical work, a balance would be needed between increasing the number of food-choice factors to achieve greater coverage without making the Case 1 BWS task too burdensome for participants.

Measures of actual food were not part of the present research, and it is a limitation that we are unable to ascertain whether the relative importance attached to various food choice motives predicts behavioral adherence to dietary patterns.

#### 5.2.2. Research Methodology

The decision to measure the importance of daily food choice motives using Case-1 BWS was both a strength and a limitation of this research. The same would be true if rating scales had been used. While such scales provide respondents with a direct mechanism for expressing absolute levels of importance, when many motives are considered important, they offer limited ability to differentiate underlying priorities or to identify the trade-offs consumers make between food choice motives (e.g., [36]). Thus, neither method is without disadvantages, and in light of the focus on diet group comparisons in the present, BWS was a sound methodological choice, despite the risk of hindering meaningful interpretation. For instance, Auger et al. [72] emphasized how a factor might seem to have low importance, not because it lacks behavioral relevance, but because the design forces respondents to trade it off against attributes that are universally valued or more salient. Therefore, lower-ranked factors cannot be interpreted as unimportant per se. Instead, they are simply less important than the other factors included in the BWS choice sets.

This limitation is pertinent to the present research, where food choice motives relating to taste-Healthiness, Freshness, and Affordability—occupied the top end of the relative spectrum, compressing the apparent importance of secondary factors such as Environmental Friendliness, Animal Welfare, Weight Control, or Familiarity (Figure 2). Without absolute benchmarks, it is not possible to determine whether these lower-ranked motives meaningfully influence daily food choices or are largely irrelevant. Anchored BWS [73,74,75], which supplements the comparative BWS tasks with one or more absolute rating or threshold questions, could have been used to mitigate this problem. The anchoring factors enable mapping individual-level BWS responses onto an interpretable absolute importance scale, thereby distinguishing genuinely unimportant factors from those that are important but overshadowed by stronger drivers. Despite these advantages, anchored BWS remains less widely adopted than the conventional (i.e., relative-only) format. This influenced the decision to forego anchoring in this research. Moreover, anchoring requires additional survey components, which adds to the respondent burden, and the analytical procedures are more technically demanding than the simple count-based strategy used in this and most other Case-1 BWS studies.

## 6. Conclusions

This is the first investigation that provides a fine-grained examination of the relative importance of a standard set of food choice motives that encompasses a wide range of reasons for various meat- or plant-based dietary habits (i.e., the nine dietary groups). The present data set provides a fine-grained examination of the relative importance of a standard set of food choice motives that covers the range of possible reasons for maintaining or adopting different diets that vary primarily in the extent to which meat is included. What emerges is that different dietary approaches are distinguished by a relatively small number of factors, among which the Sensory Appeal of food and concern for Animal Welfare are the most important. In this regard, Omnivores can be seen as opposites to vegans in terms of their motives. But other vegetarian groups are also strongly motivated by Animal Welfare, though this is mostly not evident among those who merely restrict their intake of animal proteins by adopting a flexitarian diet. The implications of these findings for encouraging dietary changes for both health and sustainability reasons suggest that increasing the perceived sensory appeal of a partly or wholly vegetarian diet may be a worthwhile strategy, as would raising awareness of animal welfare issues more broadly. A detailed analysis of participant characteristics in the various diet groups could deliver knowledge that will advance the effectiveness of such strategies.

## Figures and Tables

**Table 1:** Overview of the four studies included in the research.

Study	N (%)	Dietary Groups
1	1365 (26.7)	All dietary groups allowed
2	1330 (26.0)	Vegetarians and vegans excluded
3	1096 (21.4)	Vegans excluded
4	1320 (25.8)	Vegetarians and vegans excluded

**Table 2:** Classification scheme for the nine diet groups used in this research. Participants were shown the statements in the second column and asked to select the one that best represented their dietary habits. The final two columns show the number of consumers in each diet group in the final sample (*n* = 5111) and their percentages.

Diet Group	Statements	N	%
Omnivore	I regularly eat red meat, fish, and chicken.	3221	63.0
Flexitarian (meat reducer)	I consciously reduce meat intake, but eat meat now and then.	793	15.5
Flexitarian (red meat avoider)	I do not eat red meat, but I eat fish, chicken, and other poultry.	175	3.4
Flexitarian (pescatarian)	I do not eat red meat or chicken, but I eat fish and shellfish.	105	2.1
Flexitarian (organic/local)	I eat organic and locally grown foods, with a great overlap with foods consumed in a vegetarian diet, yet also including certain kinds of meat.	89	1.7
Vegetarian (lacto-ovo)	I do not eat meat or fish, but I eat eggs and dairy products.	450	8.8
Vegetarian (lacto)	I do not eat meat, fish, or eggs, but I eat dairy products.	90	1.8
Vegetarian (ovo)	I do not eat meat, fish, or dairy products, but I eat eggs.	42	0.8
Vegan	I do not eat meat, and I do not use products of animal origin.	146	2.9

**Table 3:** Summary of participant (UK adults) characteristics (%) in the aggregate sample (*n* = 5111) and by self-declared diet group (*n* = 9). *p*-values refer to the comparison of the nine diet groups by participant characteristic.

Characteristic	Overall	Omnivore ^1^	Flexitarian, Meat Reducer	Flexitarian, Red Meat Avoider	Flexitarian, Pescatarian	Flexitarian, Organic/Local	Vegetarian, Lacto-Ovo	Vegetarian, Lacto	Vegetarian, Ovo	Vegan	*p*-Value ^6^
N (%)	5111 (100%)	3221 (63.0%)	793 (15.5%)	175 (3.4%)	105 (2.1%)	89 (1.7%)	450 (8.8%)	90 (1.8%)	42 (0.8%)	146 (2.9%)	
Gender (%)											<0.001
Woman	53.5	49.6	59.0	68.6	71.4	57.3	63.3	61.1	69.0	35.6	
Man	46.1	50.2	40.4	31.4	27.6	42.7	35.6	38.9	28.6	63.0	
Other	0.4	0.2	0.6	0	1.0	0	1.1	0	2.4	1.4	
Age Group (%)											<0.001
18–45 y.o.	43.4	43.2	40.0	39.4	36.2	59.6	45.6	44.4	47.6	58.2	
46–69 y.o.	56.6	56.8	60.0	60.6	63.8	40.4	54.4	55.6	52.4	41.8	
Household Size (%)											0.012
1–2 people	56.6	55.6	58.5	60.0	73.3	40.4	56.0	61.1	66.7	56.8	
3–4 people	36.5	37.1	34.3	33.1	22.9	47.2	38.9	35.6	19.0	39.0	
5 or more people	6.7	7.1	6.6	6.9	2.9	12.4	4.9	3.3	14.3	4.1	
Prefer not to answer	0.3	0.2	0.6	0	1.0	0	0.2	0	0	0	
Education ^2^ (%)											<0.001
Higher educational attainment	58.6	54.9	64.7	56.0	74.3	73.0	65.8	57.8	69.0	65.8	
Lower educational attainment	38.6	42.0	32.5	41.1	24.8	24.7	31.3	41.1	31.0	32.9	
Other	1.9	2.1	1.6	1.7	0	1.1	2.2	0	0	0.7	
Prefer not to answer	0.9	0.9	1.1	1.1	1.0	1.1	0.7	1.1	0	0.7	
Employment (%)											<0.001
Working full time (≥30 h per week)	51.1	51.8	48.5	40.6	43.8	67.4	52.7	44.4	52.4	57.5	
Working part-time (<30 h per week)	16.8	15.7	17.8	22.3	23.8	11.2	18.4	23.3	11.9	21.9	
Unpaid work/home duties	5.7	5.3	6.2	8.0	2.9	3.4	8.2	8.9	7.1	2.1	
Student	2.7	2.7	1.9	3.4	0	2.2	3.3	6.7	7.1	1.4	
Unemployed	6.3	6.4	5.8	9.1	6.7	3.4	5.6	5.6	4.8	9.6	
Retired	14.5	15.3	16.9	14.9	17.1	6.7	9.6	6.7	9.5	6.8	
Other	1.9	2.0	2.0	1.1	1.0	3.4	1.3	3.3	4.8	0	
Prefer not to answer	0.9	0.7	0.9	0.6	4.8	2.2	0.9	1.1	2.4	0.7	
Food Neophobia Score ^3^	33.2 (11.8)	32.8 (12.0)	31.7 (11.3)	36.9 (11.4)	31.5 (11.8)	30.7 (10.2)	35.7 (11.2)	39.7 (12.0)	38.9 (10.8)	34.4 (10.8)	<0.001
Overall General Health ^4^	3.0 (1.1)	3.0 (1.1)	2.9 (1.2)	3.1 (1.1)	2.9 (1.2)	3.0 (1.3)	3.2 (1.1)	2.8 (1.1)	3.2 (1.2)	2.4 (1.1)	<0.001
Missing data/Prefer not to answer (%)	26.0	31.0	32.0	33.0	7.6	15.0	1.3	2.2	2.4	1.4	
Life Satisfaction ^5^	6.6 (2.3)	6.6 (2.3)	6.6 (2.3)	6.8 (2.1)	6.5 (2.3)	7.1 (1.9)	6.6 (2.2)	5.8 (2.5)	6.3 (2.4)	6.5 (2.4)	0.021
Prefer not to answer (%)	0.2	<0.1	0.1	0.0	0.0	0.0	0.2	3.3	2.4	0.0	

Table notes. ^1^ Diet groups: Omnivore (I regularly eat red meat, fish, and chicken); flexitarian, meat reducer (I consciously reduce meat intake but eat meat now and then); flexitarian, red meat avoider (I do not eat red meat but eat fish and poultry); flexitarian, pescatarian (I do not eat red meat or chicken but eat fish and shellfish); flexitarian, organic/local (I primarily eat organic and locally grown foods, sometimes including certain kinds of meat); vegetarian, lacto-ovo (I do not eat meat or fish but eat eggs and dairy products); vegetarian, lacto (I do not eat meat, fish, or eggs but eat dairy products); vegetarian, ovo (I do not eat meat, fish, or dairy products but eat eggs); vegan (I do not eat meat or use products of animal origin) [32]. ^2^ Education: Lower educational attainment (“Lower than high school”, “GCSE’s”, “A-levels”, or “Associate/technical”) and Higher educational attainment (“College” or “Postgraduate”). ^3^ Individual Food Neophobia Scores (FNS) presented as Mean (Standard Deviation) were calculated by aggregating responses across the 10 items from the Food Neophobia [44] scale rated with a fully labeled 7-point Likert scales (1 = ‘disagree strongly’ to 7 = ‘agree strongly’). ^4^ Overall general health [45] was collected on a 5-point category scale (1 = ‘Poor’, 2 = ‘Fair’, 3 = ‘Good’, 4 = ‘Very good’ and 5 = ‘Excellent’ presented as Mean (Standard Deviation). Missing/Prefer not to answer indicates the proportion of missing data from Study 4 and the number of participants who responded ‘Prefer not to answer’ in any study. ^5^ Self-rated life satisfaction [46] presented as Mean (Standard Deviation). The measure was collected on a 10-point category scale (1 = ‘Not satisfied at all’ to 10 = ‘extremely satisfied’). Prefer not to answer indicates the proportion of participants who selected ‘Prefer not to answer’. ^6^ Pearson’s Chi-squared tests with simulated *p*-value (based on 2000 replicates); One-way Analysis of Variance.

**Table 4:** Importance of 11 food choice motives in the aggregate sample (5111 UK adults), elicited using Case-1 Best–Worst scaling, and shown as mean Best-Worst (B-W) scores and associated standard deviations (SD). For mean scores, motives with different letters are statistically significant at the 5% level, based on Tukey’s Honest Significant Difference test. The final column shows relative importance scores calculated per Equation (3), giving the value 100 to the most important motive (Sensory Appeal).

Food Choice Motive	Mean (B-W)	SD (B-W)	Relative Importance
Sensory Appeal	2.2 a	2.1	100
Health	1.3 b	2.0	57
Freshness	1.1 bc	1.7	55
Price	1.0 c	2.3	43
Natural Content	0.3 d	1.7	31
Animal Welfare	−0.2 e	2.3	21
Convenience	−0.4 f	2.5	21
Env. Friendliness	−1.0 g	2.1	14
Familiarity	−1.0 g	2.0	13
Weight Control	−1.4 h	2.6	13
Social Justice	−1.8 i	2.1	9

**Table 5:** Mean Best-Worst (B-W) scores for 11 food choice motives in nine diet groups (5111 UK adults), elicited using Case-1 Best–Worst scaling (BWS). Within diet groups (columns), motives with different letters are statistically significant at the 5% level, based on Tukey’s Honest Significant Difference test. The food choice motives are listed in order of the results for the aggregate sample. Bolded values indicate the maximum values in each column.

Food Choice Motive	Omnivore (63.0%)	Flexitarian, Meat Reducer (15.5%)	Flexitarian, Red Meat Avoider (3.4%)	Flexitarian, Pescatarian (2.1%)	Flexitarian: Organic/Local (1.7%)	Vegetarian: Lacto-Ovo (8.8%)	Vegetarian: Lacto (1.8%)	Vegetarian: Ovo (0.8%)	Vegan (2.9%)
Sensory Appeal	**2.6 a**	**1.8 a**	**1.9 a**	**1.9 a**	1.2 ab	1.5 b	0.9 bc	0.9 ab	0.5 c
Health	1.1 c	**1.8 a**	**1.9 a**	1.7 ab	**2.1 a**	1.2 b	1.5 ab	1.4 ab	1.3 b
Freshness	1.4 b	1.0 b	1.1 b	0.5 cd	1.0 bc	0.2 cd	0.1 c	0.2 bc	0.0 cd
Price	1.3 b	0.8 bc	0.9 bc	0.8 bcd	0.1 cd	0.6 c	0.4 c	0.5 b	−0.1 cd
Natural Content	0.2 d	0.6 c	0.3 c	0.3 cd	1.3 ab	0.2 d	0.7 bc	0.8 ab	0.5 c
Animal Welfare	−0.9 g	−0.1 d	−0.5 d	1.2 abc	−0.3 de	**2.3 a**	**2.2 a**	**2.2 a**	**3.1 a**
Convenience	−0.1 e	−1.0 e	−0.7 de	−1.3 e	−1.0 ef	−0.7 e	−1.2 d	−1.1 cd	−1.7 e
Env. Friendliness	−1.5 i	−0.3 d	−0.9 de	0.0 d	0.0 de	−0.1 d	0.1 c	−0.1 bc	1.3 b
Familiarity	−0.7 f	−1.5 f	−1.3 ef	−1.6 e	−2.0 g	−1.7 fg	−1.6 d	−1.6 d	−2.2 ef
Weight Control	−1.3 h	−1.4 ef	−1.1 def	−1.8 e	−1.5 fg	−2.1 g	−1.9 d	−2.1 d	−2.4 f
Social Justice	−2.1 j	−1.6 f	−1.7 f	−1.6 e	−0.9 def	−1.4 f	−1.1 d	−1.0 cd	−0.4 d

Table notes. Diet groups: Omnivore (I regularly eat red meat, fish, and chicken); flexitarian, meat reducer (I consciously reduce meat intake but eat meat now and then); flexitarian, red meat avoider (I do not eat red meat but eat fish and poultry); flexitarian, pescatarian (I do not eat red meat or chicken but eat fish and shellfish); flexitarian, organic/local (I primarily eat organic and locally grown foods, sometimes including certain kinds of meat); vegetarian, lacto-ovo (I do not eat meat or fish but eat eggs and dairy products); vegetarian, lacto (I do not eat meat, fish, or eggs but eat dairy products); vegetarian, ovo (I do not eat meat, fish, or dairy products but eat eggs); vegan (I do not eat meat or use products of animal origin) [32].

## Data Availability

The authors will make the data available upon request.

## Supplementary Materials

The following supporting information can be downloaded at: https://www.mdpi.com/article/10.3390/foods15040617/s1. Table S1. Summary of participant characteristics (UK adults) in the aggregate sample and by study (S). Table S2. Tables with the 11 food choice motives statements applied in the four studies (upper table) and the balanced incomplete block design of the motives applied in the best-worst scaling task (lower table). Study 1, 2, and 3, used the same motive wording, whereas Study 4 had a slightly different wording for M11. Text S1. Data quality statement for Study 1. Text S2. Data quality statement for Study 2. Text S3. Data quality statement for Study 3. Text S4. Data quality statement for Study 4. Figure S1. Proportional distributions of B-W scores for the 11 food choice motives for the overall sample of participants (*n* = 5111). Figure S2. Proportional distributions of B-W scores for the 11 food choice motives in each of the nine dietary groups. Table S3. Standard deviations of Best-Worst (BW) scores for the eleven food choice motives across nine diet groups (United Kingdom adults, 5111 in total), elicited using Case-1 Best-Worst Scaling. Table S4. Best–Worst scores for 11 food choice motives across nine diet groups (UK adults, *n* = 5111), elicited using Case-1 Best–Worst Scaling. Post-hoc letters from Tukey’s Honest Significant Difference test indicate statistically significant differences between diet groups within each motive (column). Diet groups that do not share a letter differ at the 5% significance level. Figure S3. Funnel plot of the relative importance scores for 11 food choice motives across nine diet groups (UK adults, *n* = 5111). Importance scores were elicited using Case-1 Best-Worst Scaling (BWS) and calculated as √(B/W) following Equation (3). Table S5. Estimated distribution of diet groups in the full sample (*n* = 5111) based on the percentage (%) distribution observed in Study 1. Table S6. Mean Best-Worst (BW) scores for 11 daily food choice motives for women (*n* = 54) and men (*n* = 92) in the Vegan diet group. Scores are based on Case-1 Best-Worst Scaling (BWS). Gender differences in motives (rows) are indicated with * when significant at the 5% level. Within gender groups (columns), motives with different letters differ significantly at the 5% level. Motives are ordered according to mean BW scores in the aggregate sample (UK adults, *n* = 5111). References [76,77] are cited in the supplementary materials.
