# Supplementary material for: Omnivores, Flexitarians, Vegetarians, and Vegans Attach Different Importance to Eleven Motives for Daily Food Choice Decisions: Findings from 5111 UK Adults
Source: Foods. 2026 Feb 9;15(4):617. doi: 10.3390/foods15040617 (PMC12939115; doi:10.3390/foods15040617)
Supplement: Supplementary file 1 [file foods-15-00617-s001.zip › foods-4063807-supplementary.pdf]

Table S1. Summary of participant characteristics (UK adults) in the aggregate sample and by study (S).

Characteristic	Overall	S1	S2	S3	S4	p-value[5]
N (%)	5111 (100%)	1365 (26.7%)	1330 (26%)	1096 (21.4%)	1320 (25.8%)	
**DietType[1]**						<0.001
Omnivore	63	42	76.7	57.4	75.7	
Flexitarian, meat reducer	15.5	17	15.9	9.2	18.8	
Flexitarian, red meat avoider	3.4	4.1	3.2	2	4.2	
Flexitarian, pescatarian	2.1	4.6	2.2	0.6	0.5	
Flexitarian, organic/local	1.7	2.7	2	1.2	0.9	
Vegetarian, lacto-ovo	8.8	14.4	0	23.2	0	
Vegetarian, lacto	1.8	3.1	0	4.4	0	
Vegetarian, ovo	0.8	1.5	0	2	0	
Vegan	2.9	10.7	0	0	0	
**DietTypeGr**						<0.001
Omnivore	63	42	76.7	57.4	75.7	
Flexitarian	22.7	28.4	23.3	13	24.3	
Vegetarian	11.4	18.9	0	29.6	0	
Vegan	2.9	10.7	0	0	0	
**Gender**						0.008
Woman	53.5	53.2	52.1	56.7	52.5	
Man	46.1	46.1	47.8	43.2	46.9	
Other	0.4	0.7	0.1	0.1	0.6	
**AgeGr**						0.05
18-45	43.4	40.4	43.8	45.3	44.6	
46-69	56.6	59.6	56.2	54.7	55.4	
**HouseholdSizeGr**						0.1
1-2 people	56.6	57.3	55.6	53.5	59.3	
3-4 people	36.5	35.7	37.8	39.8	33.1	
5 or more people	6.7	6.7	6.4	6.5	7.3	
Prefer not to answer	0.3	0.4	0.2	0.3	0.3	
Education2 (%)						<0.001
Higher educational attainment	58.6	59.3	61.1	58.8	55.3	
Lower educational attainment	38.6	38.3	35	38.4	42.5	
Other	1.9	0.4	2.9	2.6	1.7	
Prefer not to answer	0.9	1.9	1	0.3	0.5	
**Employment**						<0.001
Working full time (≥30 hours per week)	51.1	50	54.1	52	48.6	
Working part time (<30 hours per week)	16.8	18.1	15.7	16.9	16.7	
No-paid work/home duties	5.7	5.6	4.7	6.2	6.5	
Student	2.7	2.4	2.9	1.8	3.5	
Unemployed	6.3	7	5.3	5.4	7.5	
Retired	14.5	13.5	14	15.1	15.5	
Other	1.9	1.2	3	2.4	1.2	
Prefer not to answer	0.9	2.2	0.4	0.3	0.5	
Food Neophobia Score[2]	33.2 (11.8)	33.2 (11.7)	31.5 (11.8)	35.1 (12.2)	33.2 (11.5)	<0.001
Overall General Health[3]	3.0 (1.1)	2.7 (1.1)	3.1 (1.1)	3.2 (1.1)	NA (NA)	<0.001
Missing data/Prefer not to answer (%)	26.0	1.5	0.0	0.9	100.0	
Life Satisfaction[4]	6.6 (2.3)	6.4 (2.4)	6.8 (2.1)	6.7 (2.3)	6.6 (2.3)	<0.001
Prefer not to answer (%)	0.2	0.0	0.0	0.7	0.0	

Table notes.

[1] Diet groups: Omnivore (I regularly eat red meat, fish, and chicken); Flexitarian, meat reducer (I consciously reduce meat intake but eat meat now and then); Flexitarian, red meat avoider (I do not eat red meat but eat fish and poultry); Flexitarian, pescatarian (I do not eat red meat or chicken but eat fish and shellfish); Flexitarian, organic/local (I primarily eat organic and locally grown foods, sometimes including certain kinds of meat); Vegetarian, lacto-ovo (I do not eat meat or fish but eat eggs and dairy products); Vegetarian, lacto (I do not eat meat, fish, or eggs but eat dairy products); Vegetarian, ovo (I do not eat meat, fish, or dairy products but eat eggs); Vegan (I do not eat meat or use products of animal origin) (De Backer, et al., 2015)

[2] Education: Lower educational attainment ("Lower than high school", "GCSE's", "A-levels", or "Associate/technical") and Higer educational attainment ("College" or "Postgraduate").

[3] Individual Food Neophobia Scores (FNS) presented as Mean (Standard Deviation) were calculated by aggregating responses across the 10 items from the Food Neophobia (Pliner, et al., 1992) scale rated with a fully labelled 7-point Likert scales (1='disagree strongly' to 7='agree strongly').

[4] Overall general health (Ware, et al., 1996) was collected on a 5-point category scale (1='Poor', 2='Fair', 3='Good', 4='Very good' and 5='Excellent' presented as Mean (Standard Deviation). Missing/Prefer not to answer indicates the proportion of missing data from Study 4 and the number of participants who responded 'Prefer not to answer' in any study.

[5] Self-rated life satisfaction (Diener, et al., 2013) presented as Mean (Standard Deviation). The measure was collected on a 10-point category scale (1='Not satisfied at all' to 10='extremely satisfied'). Prefer not to answer indicates the proportion of participants who selected 'Prefer not to answer'.

[6] Pearson's Chi-squared tests with simulated p-value (based on 2000 replicates).

Table S2. Tables with the 11 food choice motives statements applied in the four studies (upper table) and the balanced incomplete block design of the motives applied in the best-worst scaling task (lower table). Study 1, 2, and 3, used the same motive wording, whereas Study 4 had a slightly different wording for M11.

Food Choice Motive	FCQ11 (S1, S2, S3)	FCQ11 (S4)
M1	Is healthy	Is healthy
M2	Is fresh	Is fresh
M3	Is convenient (in buying and preparing)	Is convenient (in buying and preparing)
M4	Is tasty	Is tasty
M5	Is natural	Is natural
M6	Is affordable	Is affordable
M7	Helps me control my weight	Helps me control my weight
M8	Is familiar	Is familiar
M9	Is environmentally friendly	Is environmentally friendly
M10	Is animal friendly	Is animal friendly
M11	Is socially sustainable (e.g., fair-trade)	Is fair-trade

Choice set no.	Five statements per table				
1	M1	M2	M3	M5	M8
2	M2	M3	M4	M6	M9
3	M3	M4	M5	M7	M10
4	M4	M5	M6	M8	M11
5	M5	M6	M7	M9	M1
6	M6	M7	M8	M10	M2
7	M7	M8	M9	M11	M3
8	M8	M9	M10	M1	M4
9	M9	M10	M11	M2	M5
10	M10	M11	M1	M3	M6
11	M11	M1	M2	M4	M7

Text S1. Data quality statement for Study 1.

1. Participants were recruited by telephone, social media, or had self-registered with Norstat because they were interested in taking part in online research studies.
2. Norstat is ISO-certified (20252:2019) and a member of ESOMAR and the Market Research Society.
3. A total of **3490** people entered the survey, achieved by reaching out to approximately **14400**.
4. **147** people started the survey but did not complete it.
5. During pre-screening **1407** people were terminated with the following reasons:
    - 5a. **18** did not meet the English proficiency criteria.
    - 5b. **18** did not meet the age range criteria.
    - 5c. **72** did not provide consent.
    - 5d. **1229** were not considered as their responses exceeded the predefined participant quota for their demographic group (i.e., overquota).
    - 5e. **70** provided inconsistent responses, falling into an illogical trap regarding their dietary style by indicating a vegetarian/vegan diet (PS6_ALL) while reporting meat (in Q05_ALL_1) or fish (in Q05_ALL_2) consumption.
6. During survey completion, people were terminated with the following reasons:
7. Of the **1936** people who completed the survey, **571** people were removed *post-hoc* with the following reasons:
    - 7a. **44** due to a response scale error that caused scale compression in D07_ALL (Life Satisfaction) during the soft launch.
    - 7b. **36** had completed the survey very quickly (operationalised as faster than one-third of the median response time), which is linked to careless responding (Goldammer, Annen, Stöckli, & Jonas, 2020).
    - 7c. **190** were flatlining in their responses (operationalised as 'range=1', i.e., meaning they selected only adjacent answer options for all statements) in the 10-item food neophobia scale (Q07_ALL) (Schonlau, et al., 2015)
    - 7d. **301** people had a normalised error variance index below 0.5 for the task about food choice motives (Case 1 Best-Worst Scaling), indicating a low response consistency (Llobell et al., 2025).
8. In total, **1365** people were included for analysis.
9. There were no mindfulness exercises prior to starting the survey, and gamification was not used.
10. The data was checked for duplicate IP addresses but none were found.
11. The median time to complete the entire survey was **14** minutes (IQR = **9.4**).
12. The survey was completed by **781** people using their smartphones, **53** using tablets, and **838** on desktops or laptops.
13. Of the **1365** participants, **1566** were recruited directly by Norstat, while the remaining **106** were provided by Norstat's partners (ISO-certified and ESOMAR members) to meet the quota for participants with a vegetarian diet.

Text S2. Data quality statement for Study 2.

1. Participants were recruited by telephone, social media, or had self-registered with Norstat because they were interested in taking part in online research studies.
2. Norstat is ISO-certified (20252:2019) and a member of ESOMAR and the Market Research Society.
3. A total of **2790** people entered the survey, achieved by reaching out to approximately **24529**.
4. **339** people started the survey but did not complete it.
5. Of those people entering the survey, **647** people were terminated with the following reasons:
   - 5a. **11** did not meet the English proficiency criteria
   - 5b. **96** did not meet the age range criteria
   - 5c. **14** did not provide consent
   - 5d. **4** were not considered as their responses exceeded the predefined participant quota for their demographic group (i.e., overquota)
   - 5e. **133** were veg*n and did not fit into the predefined diet type quota
   - 5f. **136** did not provide two of the relevant eating occasions (Breakfast, AM snack, Lunch, PM snack, Dinner,Evening snack).
   - 5g. **177** provided inconsistent response, indicationg two days (for eating occasion 1 and 2) that are not consecutive (e.g., Sunday and Tuesday) in the 24-hour food recall.
   - 5h. **13** provided inconsistent response, falling into an illogical trap regarding their dietary style by indicating a flexitarian diet with no red meat consumption yet choosing red meat as a food item in the 24-hour food recall.
   - 5i. **1** provided inconsistent response, falling into an illogical trap regarding their dietary style by indicating a flexitarian diet with no red meat and chicken consumption yet choosing red meat or chicken as a food item in the 24-hour food recall.
   - 5j. **62** provided inconsistent response, failing an attention check applied to Q01, indicating ‘None of the above’ across all food categories, as this would indicate no foods present in the eating occasion.
6. Of the **1804** people who completed the survey, **474** people were removed *post-hoc* with the following reasons:
   - 6a. **136** were part of the softlaunch, which had an error.
   - 6b. **29** were found to have a duplicate ip-addresses.
   - 6c. **15** had completed the survey very quickly (operationalised as faster than one-third of the median response time), which is linked to careless responding (Goldammer, Annen, Stöckli, & Jonas, 2020).
   - 6d. **92** were flatlining in their responses (operationalised as ‘range=1’, i.e., meaning they selected only adjacent answer options for all statements) in the 10-item food neophobia scale (Schonlau, et al., 2015).
   - 6e. **202** people had a normalised error variance index below 0.5 for the task about food choice motives (Case 1 Best-Worst Scaling), indicating a low response consistency (Llobell et al., 2025).
7. In total, **1330** people were included for analysis.
8. There were no mindfulness exercises prior to starting the survey, and gamification was not used.

9. The median time to complete the entire survey was **18.4** minutes (IQR = **11.2**).
10. The survey was completed by **605** people using their smartphones, 0 using tablets, and **624** on desktops or laptops.

Text S3. Data quality statement for Study 3.

Participants were recruited by telephone, social media, or had self-registered with Norstat because they were interested in taking part in online research studies.

1. Norstat is ISO-certified (20252:2019) and a member of ESOMAR and the Market Research Society.
2. A total of **4627** people entered the survey, achieved by reaching out to approximately **10700**.
3. **500** people started the survey but did not complete it.
4. Of those people entering the survey, **2874** people were terminated with the following reasons:

    - 5a. **18** did not meet the English proficiency criteria
    - 5b. **26** did not meet the age range criteria (18-69 years old)
    - 5c. **1536** did not fit the quota of having either low willingness to transisiton to vegetarian diet, or were already vegetarian
    - 5d. **107** did not provide informed consent
    - 5e. **163** exceeded the predefined participant quota for their demographic group (i.e., overquota)
    - 5f. **622** were vegan and did not fit into the predefined diet type quota
    - 5g. **402** failed the theory of mind pre-screening check

5. Of the **1253** people who completed the survey, **157** people were removed *post-hoc* with the following reasons:

    - 6a. **21** had completed the survey very quickly (operationalised as faster than one-third of the median response time), which is linked to careless responding (Goldammer, Annen, Stöckli, & Jonas, 2020).
    - 6b. **33** were flatlining in their responses (operationalised as ‘range=1’, i.e., , meaning they selected only adjacent answer options for all statements) in the 10-item food neophobia scale (Schonlau, et al., 2015).
    - 6c. **103** people had a normalised error variance index below 0.5 for the task about food choice motives (Case 1 Best-Worst Scaling), indicating a low response consistency (Llobell et al., 2025).

6. In total, **1096** people were included for analysis.
7. There were no mindfulness exercises prior to starting the survey, and gamification was not used.
8. The data was checked for duplicate IP addresses but none were found.
9. The median time to complete the entire survey was **17.4** minutes (IQR = **14.8**).

Text S4. Data quality statement for Study 4.

1. Participants were recruited by telephone, social media, or had self-registered with Norstat because they were interested in taking part in online research studies.
2. Norstat is ISO-certified (20252:2019) and a member of ESOMAR and the Market Research Society.
3. A total of **2287** people entered the survey
4. **138** people started the survey but did not complete it.
5. Of those people entering the survey, **416** people were terminated with the following reasons:
   - 5a. **45** did not eat meat as per study quota
   - 5b. **24** did not eat meat or dairy as per study quota
   - 5c. **347** were not considered as their responses exceeded the predefined participant quota for their demographic group (i.e., overquota)
6. Of the **1733** people who completed the survey, **413** people were removed *post-hoc* with the following reasons:
   - 6a. **66** were part of the soft-launch, which had an error.
   - 6b. **8** had completed the survey very quickly (operationalised as faster than one-third of the median response time), which is linked to careless responding (Goldammer, Annen, Stöckli, & Jonas, 2020).
   - 6c. **144** were flatlining in their responses (operationalised as 'range=1', i.e., , meaning they selected only adjacent answer options for all statements) in the 10-item food neophobia scale (Schonlau, et al., 2015).
   - 6d. **195** people had a normalised error variance index below 0.5 for the task about food choice motives (Case 1 Best-Worst Scaling), indicating a low response consistency (Llobell et al., 2025).
7. In total, **1320** people were included for analysis.
8. There were no mindfulness exercises prior to starting the survey, and gamification was not used.
9. The data was checked for duplicate IP addresses but none were found.
10. The median time to complete the entire survey was **0.3** minutes (IQR = **0.2**).

Figure S1. Proportional distributions of B-W scores for the 11 food choice motives for the overall sample of participants (N=5,111).

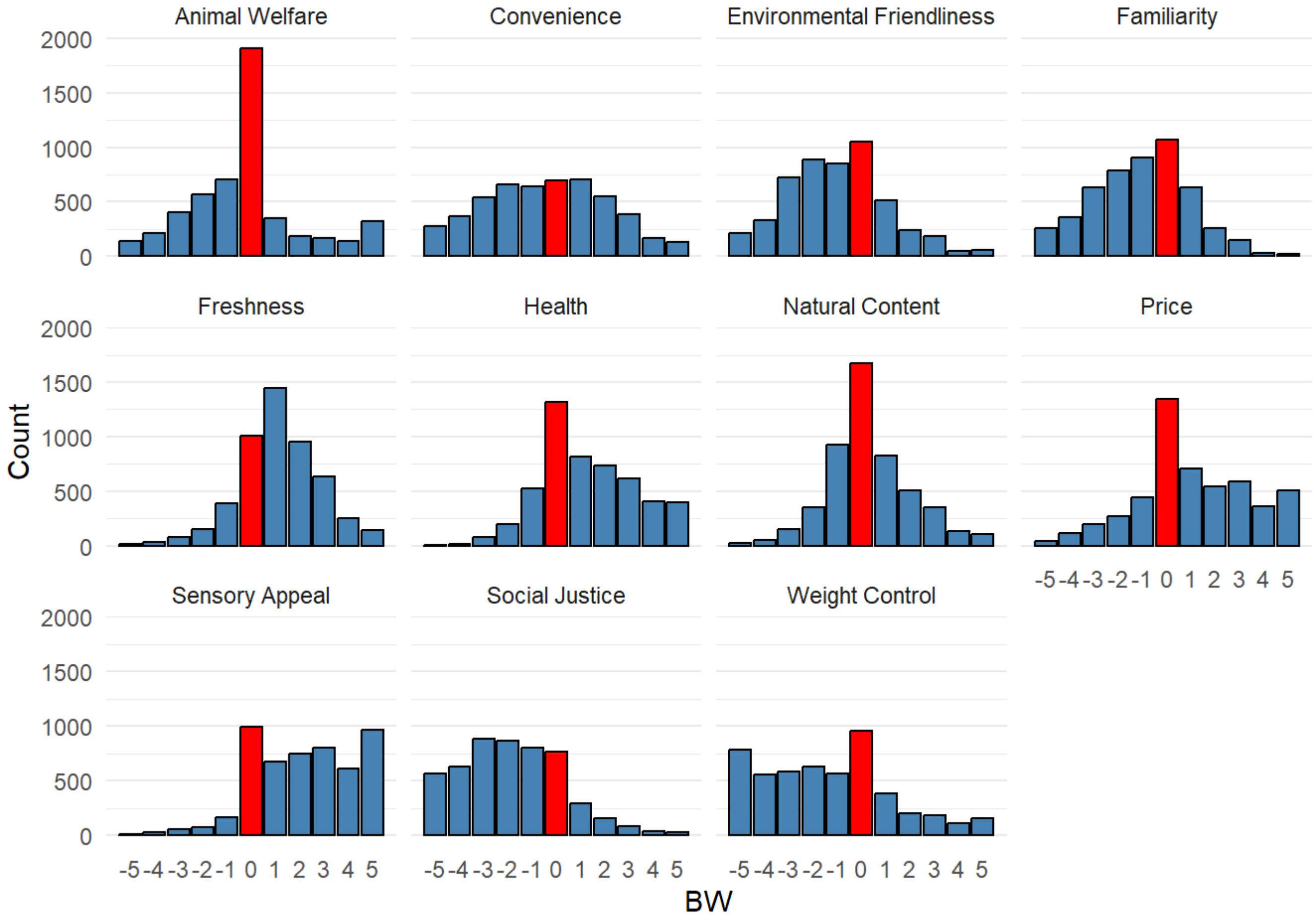

Figure S2. Proportional distributions of B-W scores for the 11 food choice motives in each of the nine dietary groups.

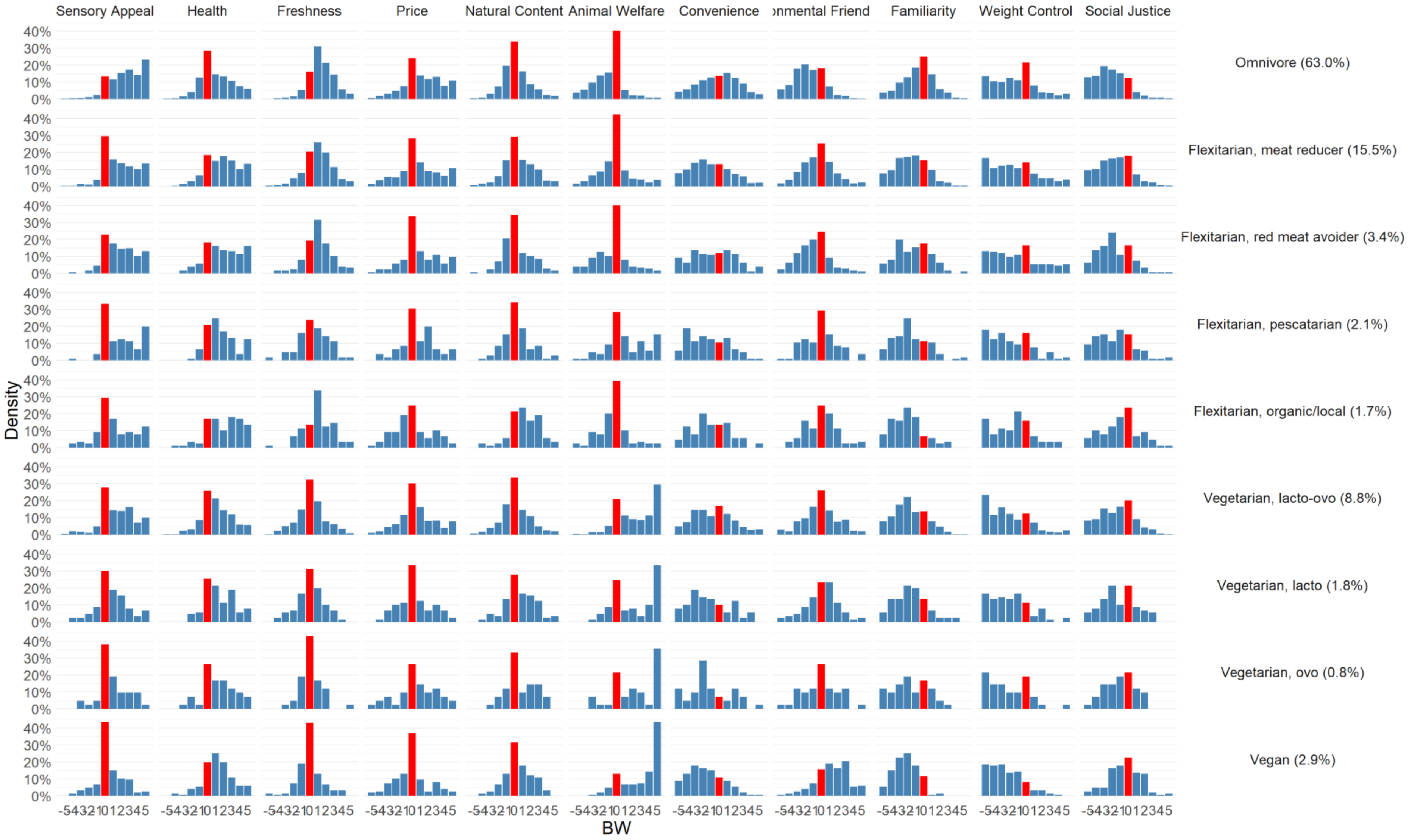

Table S3. Standard deviations of Best-Worst (BW) scores for the eleven food choice motives across nine diet groups (United Kingdom adults, 5,111 in total), elicited using Case-1 Best-Worst Scaling.

**Food Choice Motive**	Overall	Omnivore (63.0%)	Flexitarian, meat reducer (15.5%)	Flexitarian, red meat avoider (3.4%)	Flexitarian, pescatarian (2.1%)	Flexitarian, organic/local (1.7%)	Vegetarian, lacto-ovo (8.8%)	Vegetarian, lacto (1.8%)	Vegetarian, ovo (0.8%)	Vegan (2.9%)
Sensory Appeal	2.1	2.0	2.0	1.9	2.1	2.3	2.1	2.0	1.8	1.7
Health	2.0	1.9	2.0	2.1	1.8	2.1	1.9	1.8	2.0	1.8
Freshness	1.7	1.6	1.8	1.7	1.9	1.8	1.8	1.6	1.4	1.5
Price	2.3	2.3	2.4	2.2	2.1	2.3	2.2	2.0	2.4	2.1
Natural Content	1.7	1.7	1.9	1.7	1.7	1.8	1.8	1.9	1.8	1.7
Animal Welfare	2.3	1.8	1.9	2.1	2.4	1.8	2.3	2.5	2.6	2.2
Convenience	2.5	2.5	2.5	2.7	2.4	2.3	2.4	2.5	2.5	2.2
Env. Friendliness	2.1	1.9	2.0	2.0	2.0	2.0	2.1	2.0	2.2	2.1
Familiarity	2.0	1.9	2.0	2.1	2.1	1.9	2.0	2.0	2.2	1.5
Weight Control	2.6	2.6	2.8	2.9	2.5	2.4	2.5	2.4	2.4	2.1
Social Justice	2.1	2.0	2.1	2.0	2.3	2.2	2.1	2.1	1.8	2.0

Table S4. Best–Worst scores for 11 food choice motives across nine diet groups (UK adults, N = 5,111), elicited using Case-1 Best–Worst Scaling. Post-hoc letters from Tukey's Honest Significant Difference test indicate statistically significant differences between diet groups within each motive (column). Diet groups that do not share a letter differ at the 5% significance level.

DietType	Sensory Appeal	Health	Freshness	Price	Natural Content	Animal Welfare	Convenience	Env. Friendliness	Familiarity	Weight Control	Social Justice
Omnivore (63.0%)	2.6 A	1.1 B	1.4 A	1.3 A	0.2 C	-0.9 E	-0.1 A	-1.5 D	-0.7 A	-1.3 A	-2.1 D
Flexitarian, meat reducer (15.5%)	1.8 B	1.8 A	1.0 B	0.8 B	0.6 B	-0.1 D	-1.0 B	-0.3 B	-1.5 B	-1.4 A	-1.6 C
Flexitarian, red meat avoider (3.4%)	1.9 B	1.9 A	1.1 AB	0.9 AB	0.3 BC	-0.5 DE	-0.7 B	-0.9 C	-1.3 B	-1.1 A	-1.7 CD
Flexitarian, pescatarian (2.1%)	1.9 B	1.7 AB	0.5 BCD	0.8 ABC	0.3 BC	1.2 C	-1.3 BC	0.0 B	-1.6 BC	-1.8 ABC	-1.6 BCD
Flexitarian, organic/local (1.7%)	1.2 BCD	2.1 A	1.0 ABC	0.1 CD	1.3 A	-0.3 DE	-1.0 BC	0.0 B	-2.0 BC	-1.5 AB	-0.9 AB
Vegetarian, lacto-ovo (8.8%)	1.5 BC	1.2 B	0.2 D	0.6 BC	0.2 C	2.3 B	-0.7 B	-0.1 B	-1.7 BC	-2.1 BC	-1.4 BC
Vegetarian, lacto (1.8%)	0.9 CD	1.5 AB	0.1 CD	0.4 BCD	0.7 ABC	2.2 B	-1.2 BC	0.1 B	-1.6 BC	-1.9 ABC	-1.1 ABC
Vegetarian, ovo (0.8%)	0.9 BCD	1.4 AB	0.2 BCD	0.5 ABCD	0.8 ABC	2.2 ABC	-1.1 BC	-0.1 BC	-1.6 ABC	-2.1 ABC	-1.0 ABC
Vegan (2.9%)	0.5 D	1.3 AB	0.0 D	-0.1 D	0.5 ABC	3.1 A	-1.7 C	1.3 A	-2.2 C	-2.4 C	-0.4 A

Figure S3. Funnel plot of the relative importance scores for 11 food choice motives across nine diet groups (UK adults, n = 5,111). Importance scores were elicited using Case-1 Best-Worst Scaling (BWS) and calculated as √(B/W) following Equation 3.

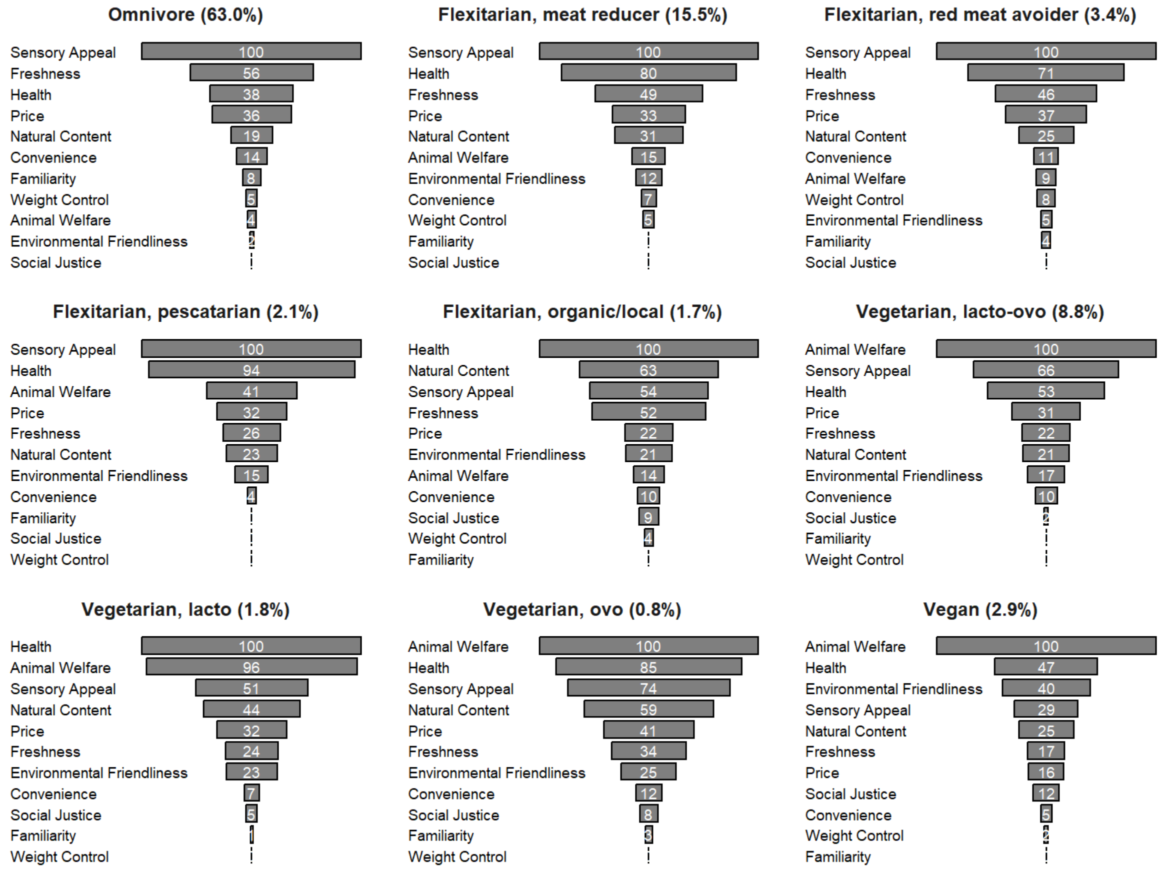

Table S5. Estimated distribution of diet groups in the full sample (N = 5,111) based on the percentage (%) distribution observed in Study 1.

Diet group (Study 1)	%	Expected N for N=5,111
Omnivore	42.0	2147
Flexitarian, meat reducer	17.0	869
Flexitarian, red meat avoider	4.1	210
Flexitarian, pescatarian	4.6	235
Flexitarian, organic/local	2.7	138
Vegetarian, lacto-ovo	14.4	736
Vegetarian, lacto	3.1	158
Vegetarian, ovo	1.5	77
Vegan	10.7	547

Table S6. Mean Best-Worst (BW) scores for 11 daily food choice motives for women (n=54) and men (n=92) in the *Vegan* diet group. Scores are based on Case-1 Best-Worst Scaling (BWS). Gender differences in motives (rows) are indicated with * when significant at the 5% level. Within gender groups (columns), motives with different letters differ significantly at the 5% level. Motives are ordered according to mean BW scores in the aggregate sample (UK adults, N = 5,111).

Daily Food Choice Motive	Women (39%)	Men (63%)
Sensory Appeal	1.2* e	0.2* bc
Health	1.0 e	1.5 d
Freshness	0.0 cde	0.1 bc
Price	0.5* de	-0.4* b
Natural Content	-0.2* cd	0.9* cd
Animal Welfare	3.6* f	2.8* e
Convenience	-1.6 ab	-1.7 a
Environmental Friendliness	0.7* de	1.7* d
Familiarity	-2.1 a	-2.2 a
Weight Control	-2.2 a	-2.5 a
Social Justice	-0.7 bc	-0.3 b
